# Shrinkage and Creep Properties of Low-Carbon Hybrid Cement

**DOI:** 10.3390/ma17174417

**Published:** 2024-09-07

**Authors:** Vít Šmilauer, Lenka Dohnalová, Pavel Martauz

**Affiliations:** 1 Department of Mechanics, Faculty of Civil Engineering, Czech Technical University in Prague, Thákurova 7, 166 29 Prague, Czech Republic; lenka.dohnalova@fsv.cvut.cz; 2 Považská Cementáreň Cement Plant (PCLA), 01863 Ladce, Slovakia; martauz.p@pcla.sk

**Keywords:** creep, shrinkage, hybrid cement, H-cement, low carbon

## Abstract

Hybrid cements combine clinker with large amount of supplementary cementitious materials while utilizing hydration and alkali activation processes. This paper summarizes shrinkage and creep properties of industrially produced H-cement, containing only 20% of Portland clinker. In comparison with a reference cement CEM II/B-S 32.5 R, autogenous shrinkage is smaller after 7 days, and drying shrinkage is similar at similar times. A different capillary system of H-cement leads to faster water mass loss during drying. Basic and total creep of concrete remains in the standard deviation of B4 and EC2 creep models. The results demonstrate that shrinkage and creep properties of concrete made from H-cement have similar behavior as conventional structural concrete or high-volume fly ash concrete.

## 1. Introduction

Since the patent of Portland cement in 1824, the cement industry has developed Portland clinker to become the main ingredient of currently used blended cements. Global cement production was estimated at 4.1 Gt in 2019, emitting 0.65–0.92 tons of CO_2_/ton depending on cement plant technology and the amount of clinker in cement [1]. This corresponds to about 8% of the global CO_2_ emissions. The construction industry is determined to increase the sustainability by incorporating new materials and technologies [2]. Current concretes provide feasible solutions to several engineering projects, where improvements are sought, especially in areas of carbonation, chemical resistance, durability, deterioration and volume changes [3].

Adding KOH to ground granulated blast-furnace slag (GGBFS) was systematically studied by H. Kühl in 1930, formulating the principles of alkali activation. In 1957, V. Glukhovsky found the possibility to produce an alkali-activated binder even with calcium-free aluminosilicates (clays)—see the historical development in [4,5]. It became clear that C-S-H gel, as the main hydration product of Portland clinker, or C-A-S-H gel, which are found more in blended cements, can be substituted with N-A-S-H or N-(C)-A-S-H gels by incorporating alkalies to the system [6]. Blending Portland and alkali-activated systems leads to so-called hybrid cements, yielding a wide range of hydration products and properties inherited from both systems [6].

Coal fly ash, GGBFS, calcined mineral clays, including metakaolin, or alkali-rich solid wastes constitute common aluminosilicate precursors [7]. Alkali-activated cements are used in a niche market by dozens of companies, spanning from acid and fire protection to the construction of buildings [7]. Common activators, sodium hydroxide and sodium silicate, are the most expensive parts, increasing the concrete unit price by 2–3× when compared to its Portland counterpart [7]. Hybrid systems are price-competitive due to the fact that activators may be present in smaller amounts and in a solid form, combining both hydration and alkali activation processes.

In 2016, a hybrid cement “H-cement” was patented by Považská cementáreň, a.s., Ladce, the Slovak Republic. Its characteristic composition uses only 20% of Portland clinker, which is supplemented with fly ash, slag and a small amount of a Na_2_SO_4_ activator from alkali waters of bauxite residues. Basic properties have been summarized in other papers [8,9].

Early results have successfully demonstrated concrete production up to C30/37 strength class and with lower drying shrinkage than Portland cement concrete [8,9]. Further research proved excellent high ASR resistance [8], high resistance to sulphates, high resistance to fire, compatibility with recycled aggregates, higher adhesion [10] and approximately 40% CO_2_ emissions compared to CEM II/B-S 32.5 R due to 20% content of Portland clinker. Promising applications for thermal energy storage have been also documented, demonstrating higher thermal capacity and thermal diffusivity [11].

The main objectives of this paper are to investigate the shrinkage and creep properties of concrete made from H-cement, which contains significantly less alkalies than classical alkali-activated systems. Generally, alkali-activated slags have showed higher autogenous shrinkage [12] and higher drying shrinkage [13] than their Portland cement counterparts. On the other hand, high-volume fly ash concrete (HVFAC), replacing at least 50% of cement, was found to reduce both autogenous and drying shrinkage [14,15]. This paper provides experimental data for autogenous shrinkage, drying shrinkage accompanied with mass loss, and basic and total creep. The results demonstrate that concrete made from H-cement behaves similarly as conventional structural concrete or HVFAC.

## 2. Materials and Methods

### 2.1. H-Cement and CEM II/B-S

Table 1 shows the oxide composition of four components of H-cement, i.e., clinker, fly ash, slag and an alkali activator, as obtained from separated XRF analyses using SPECTRO X-LAB 2000, SPECTRO Analytical Instruments Inc., Kleve, Germany. Granulated blast furnace slag comes from the steel company Třinecké železárny, Třinec, the Czech Republic. However, especially in the case of slag and fly ash, their chemical composition varies over time. The characteristic composition of H-cement uses 20% clinker, 65% fly ash, 10% slag and 5% of an alkali activator, mostly Na_2_SO_4_—see Table 1. High Blaine fineness of H-cement 610 m^2^/kg promotes early-age reactivity. In addition, a reference cement CEM II/B-S 32.5 R, Mokrá, the Czech Republic, is used for comparison, having a Blaine fineness of 331 m^2^/kg. The reference cement was chosen due to slow hydration kinetics similar to H-cement, its wide availability, a lower carbon footprint with 29% clinker substitution level and available experimental data.

Strength evolution is summarized in Table 2 according to EN 196-1 [16]. In the test, H-cement used decreased w/c = 0.40.

### 2.2. Mix Design

Table 3 summarizes mix designs for mortar and concrete with the same w/c = 0.45. Mortar and concrete contains 58% and 69.1% of aggregates by volume, respectively. A reference mortar made from CEM II/B-S 32.5 R was used for the comparison.

### 2.3. Isothermal Calorimetry

Isothermal calorimetry was performed at 20 °C with the TamAIR calorimeter (Thermometric AB, Stochholm, Sweden). The pastes were mixed externally by hand for approximately 30 s and vibrated in the IKA Vortex I orbital shaker for 20 s. The procedure followed the prEN 196-11 Method “A” for external mixing with one modification; the heat released before 45 min was calculated from the known initial temperature, the estimated heat capacity and integrated heat flow up to 45 min. Calorimetry always used two samples with approximately 18 g of cement in each ampule with negligible differences.

### 2.4. Autogenous and Drying Shrinkage

**Figure 1:**
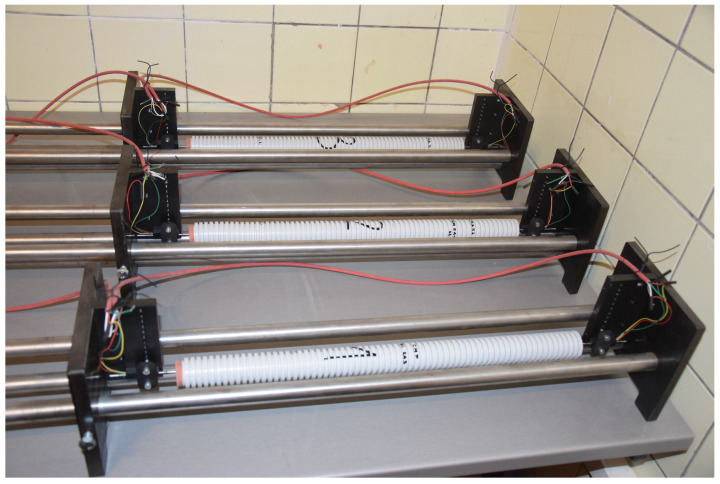
Autogenous shrinkage measurement in corrugated tubes.

Autogenous shrinkage of the cement paste followed ASTM C1698-19 [17] and took place on the horizontal corrugated tubes with an inner minimum diameter of 23.0 mm and the effective length of 400 mm. A pair of linear displacement sensors located at both ends of the sample measured the changes in length, and the values were automatically recorded at a maximum interval of 30 min—see Figure 1. The strain was zeroed at the final set, according to ASTM C1698-19, and the measurement was run for 34 days.

**Figure 2:**
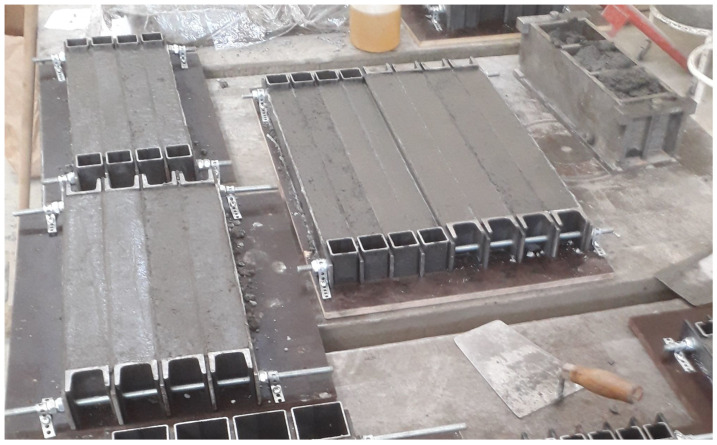
Production of H-cement prisms for drying shrinkage measurements.

**Figure 3:**
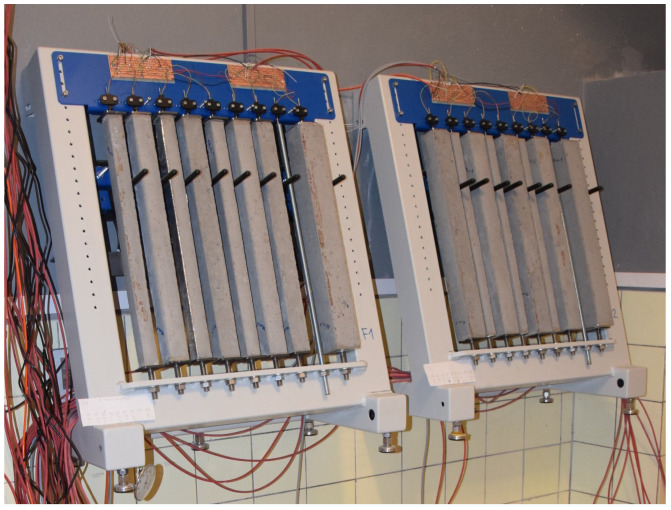
Frames for drying shrinkage experiments.

Drying shrinkage of mortars or concrete used 500 mm long prisms—see Figure 2. Mortar prisms had cross-sections of 25×25, 30×30, 40×40, and 60×60 mm, and concrete prisms used cross-sections of 40×40 and 60×60 mm. The aim was to achieve final shrinkage values on smaller specimens with regard to the maximum size of the aggregate and workability. The samples were sealed for 14 days and then exposed to drying in an environment of 21 ± 2 °C and relative humidity of 51 ± 3% for 350 days—see Figure 3.

### 2.5. Creep

**Figure 4:**
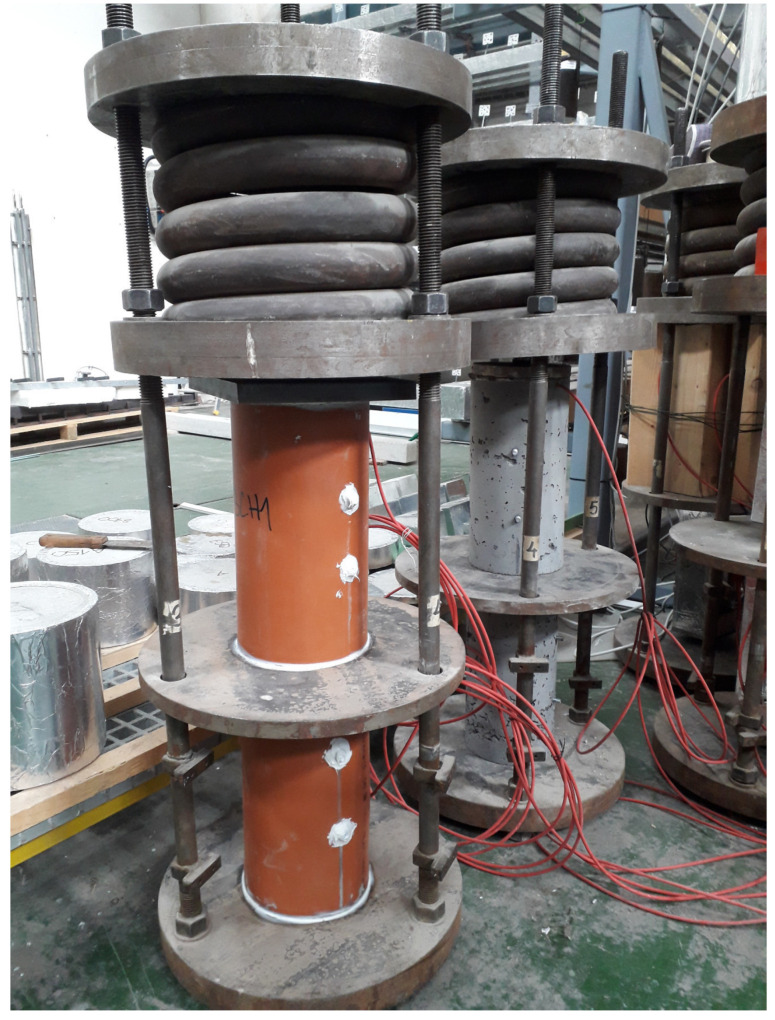
Measurement of creep of H-cement concrete cylinders in rigs.

Basic and total creep measurement took place in creep rigs—see Figure 4. A hydraulic press compressed the spring with a centric force of 100 kN, and two specimens were kept under the load for 350 days without any load compensation. A force loss of less than 2% was recorded when unloading. The samples were cylinders with a diameter of 150 mm and a height of 300 mm, and a vibrating wire strain gauge was placed inside the cylinder. The samples were loaded at 14 days of age with a stress value of −5.659 MPa, at 19% of compressive strength to recover linear creep.

Creep compliance functions in EC2 [18] and B4 [19] models describe deformations in the general form as

(1) $$\begin{array}{c} J\left(t,t^{\prime }\right)=J_{bc}\left(t,t^{\prime }\right)+J_{dc}\left(t,t^{\prime },t_{0}\right), \end{array}$$

where $J_{bc}\left(t,t^{\prime }\right)$ stands for the basic creep compliance and $J_{dc}\left(t,t^{\prime },t_{0}\right)$ for the drying creep compliance. If a unit stress is applied at loading age $t^{\prime }$, the compliance function in Equation (1) describes the evolution of stress-related strain, including the initial elastic strain. The EC2 model expresses the compliance function using a creep coefficient as

(2) $$\begin{array}{c} J\left(t,t^{\prime }\right)=\frac{1}{E(t^{\prime })}+\frac{\varphi_{28}}{E(28)}. \end{array}$$

## 3. Results and Discussion

### 3.1. Isothermal Calorimetry

**Figure 5:**
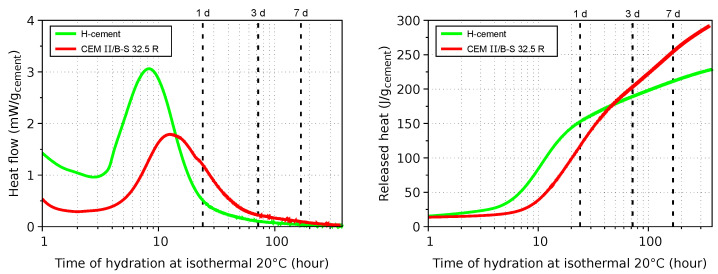
Heat flow and released heat from isothermal calorimetry.

The results from isothermal calorimetry are summarized in Figure 5. Released heat at 1 h, corresponding to the end of initial period, shows similar values for both cements, reflecting mostly the dissolution of clinker surface particles and/or the dissolution of Na_2_SO_4_ in the activator. Induction and accelerating periods up to 10 h demonstrate a higher surface area of H-cement, in the ratio of 610/331 = 1.84, which approximately corresponds to the ratio of heat flows. In addition, silicate and aluminate monomers are formed from fly ash/slag contained in H-cement. The decelerating period of H-cement encompasses the ongoing hydration of clinker, pozzolanic and latent hydraulic reactions, as well as the nucleation, growth, polymerization and polycondensation of alumosilicate monomers. Reaction products generally span across a wide range from C-S-H and C-A-S-H to N-A-S-H gels [20] and strongly depend on the pH level [4]; however, a detailed analysis of a H-cement reaction has not been performed yet. Seven-day released heat corresponds to 211 and 254 J/g for H-cement and CEM II/B-S 32.5 R, respectively.

### 3.2. Autogenous Shrinkage

**Figure 6:**
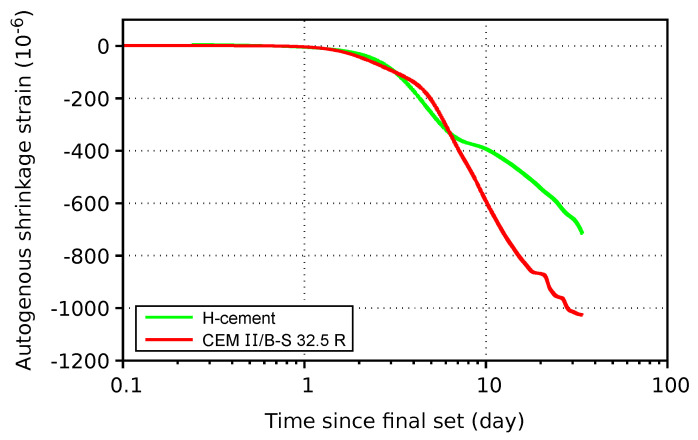
Autogenous shrinkage of pastes.

Autogenous shrinkage was zeroed at the final set, according to ASTM C1698-19 [17]. Figure 6 shows strain evolution for cement pastes made from H-cement and CEM II/B-S 32.5 R, both at w/c = 0.40. H-cement generally exhibits similar shrinkage up to 7 days and smaller shrinkage afterwards. Such a result seems contradictory to Li et al. [21], reporting that the alkali activation of fly ash or slag induces autogenous shrinkage that is several times higher against OPC pastes. However, such a conclusion was confirmed for only 25–50% clinker substitution levels, while a substitution above 50% led to a decrease due to highly diluted system with slowly reacting fly ash [22]. Poromechanics offers another explanation where the creation of capillary menisci due to self-desiccation exerts compressive stress on stiff microstructures in partially saturated porous media [23]. According to the model, higher stiffness in early age, larger curvature of menisci and lower volume shrinkage mitigate macroscopic autogenous shrinkage. Scaling the autogenous shrinkage from paste to concrete in Table 3 is possible with a factor of approximately 0.15, according to [24]. Ultimate autogenous shrinkage strain for H-cement-based concrete would attain approximately −100 · 10^−6^. Ultimate autogenous shrinkage is much closer to high-volume fly ash concrete (HVFAC) [25] than alkali-activated slag or slag/fly ash pastes [21]. In this regard, H-cement resembles a more dilute and highly blended system, which is accelerated by low alkali content.

### 3.3. Drying Shrinkage

**Figure 7:**
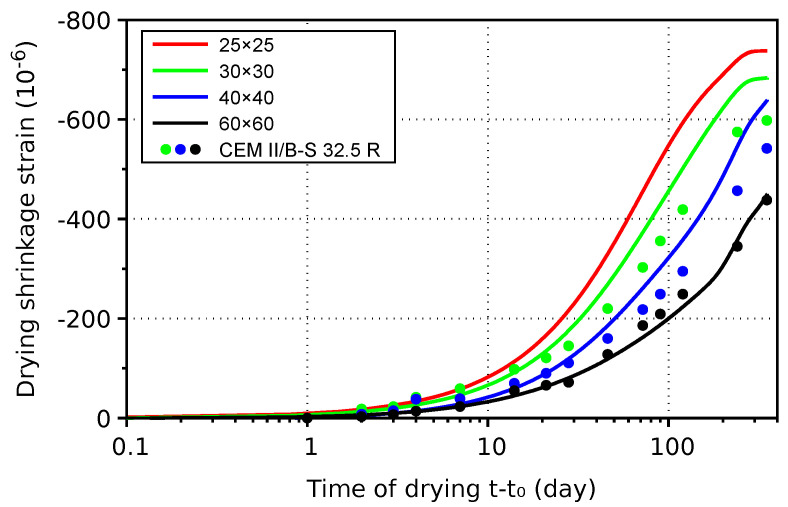
Drying shrinkage of mortars made from H-cement and CEM II/B-S 32.5 R cement.

The mortar of H-cement was subjected to drying after 14 days of sealed curing. After almost a year of drying, the ultimate value of shrinkage strain, −740 · 10^−6^, was reached on the smallest samples of 25×25 mm—see Figure 7. Drying follows the correct trend where the same deformation is achieved in the square ratio of edge size, i.e., 50×50 and 25×25 mm samples will have a fourfold difference in time for the same shrinkage values. The reproducibility is excellent, and the lines show an average from two or three prisms. Comparative values with CEM II/B-S 32.5 R cement are plotted with points, showing similar kinetics and ultimate values of mortars. Smaller samples exhibit faster drying shrinkage and slightly higher ultimate values [26]. Such a phenomenon is generally related to non-uniform moisture transport, internal stress relaxation and surface microcracking.

**Figure 8:**
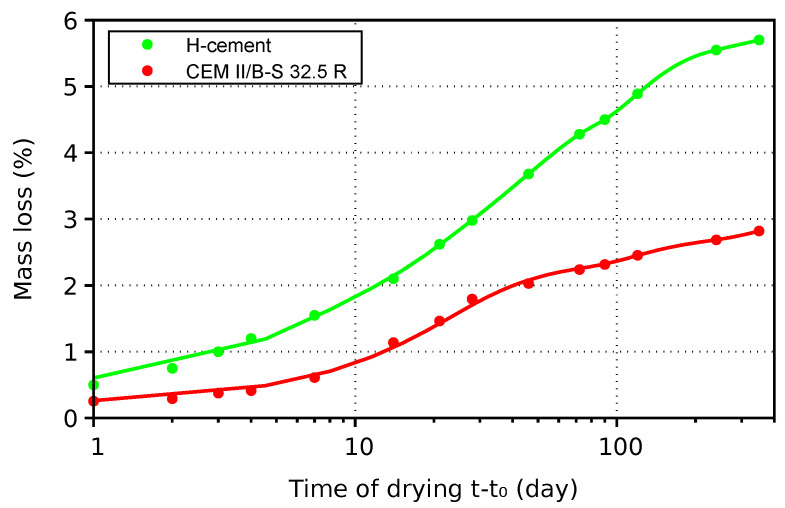
Mass loss of two mortars with cross-sections of 30×30 mm.

H-cement shows mass loss that is approximately two times higher at the same drying time—see Figure 8. A differential equation governing water mass balance of a single-fluid medium can be used to explain this, as follows:

(3) $$\begin{array}{ccc} \frac{\partial w}{\partial h}\frac{\partial h}{\partial t} & = & -\nabla \cdot J_{w}+w_{n}\frac{d\alpha }{dt}, \end{array}$$

where *h* is relative humidity (-), $\frac{\partial w}{\partial h}$ is humidity-dependent moisture capacity (kgm^−3^), $J_{w}$ (kgm^−2^s^−1^) represents the moisture flux, the sink term $w_{n}$ corresponds to non-evaporable water content for complete hydration (kgm^−3^) and α is the degree of reaction (-). The moisture flux is defined further by the constitutive law [27]:

(4) $$J_{w}=-c\left(h\right)\nabla h,$$

where c(h) (kgm^−1^s^−1^) is the moisture permeability that is dependent on relative humidity. The higher moisture loss $\frac{\partial w}{\partial t}$ is therefore controlled by higher moisture permeability and a lower rate of evaporable water binding $w_{n}\frac{d\alpha }{dt}$. Indeed, total porosity (encompassing pore diameters 0.534 nm–1.82 μm from MIP at 5 year age) yields 19.67% for H-cement-based mortar and 11.95% for OPC mortar with low C_3_A content [9].

**Figure 9:**
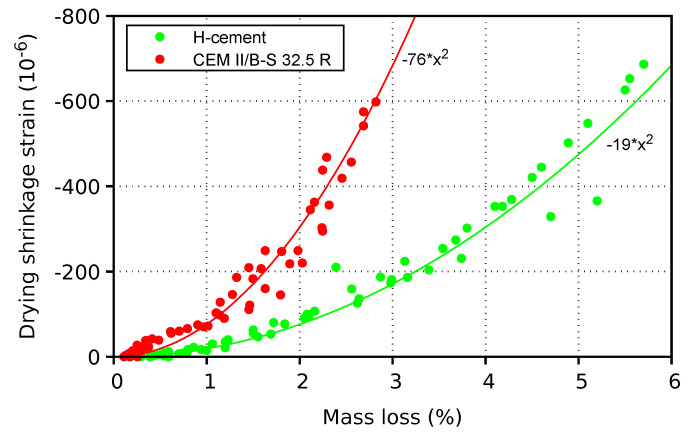
Relationship between drying shrinkage and mass loss of two mortars.

The relationship between weight loss and drying shrinkage is depicted in Figure 9, merging data from the cross-sections of 30×30, 40×40, 50×50, and 60×60 mm. The data are interpolated with a power function, showing that for the same drying shrinkage, strain H-cement loses approximately twice as much water as CEM II/B-S 32.5 R. Such a behavior can be explained with the larger emptied capillary pores of H-cement, exerting less pressure on the solid skeleton due to the lower curvature of menisci. Larger porosity with an abundance of evaporable water promotes internal curing, which can become the primary cause of drying out environments with vapor barriers, such as floors or slabs.

**Figure 10:**
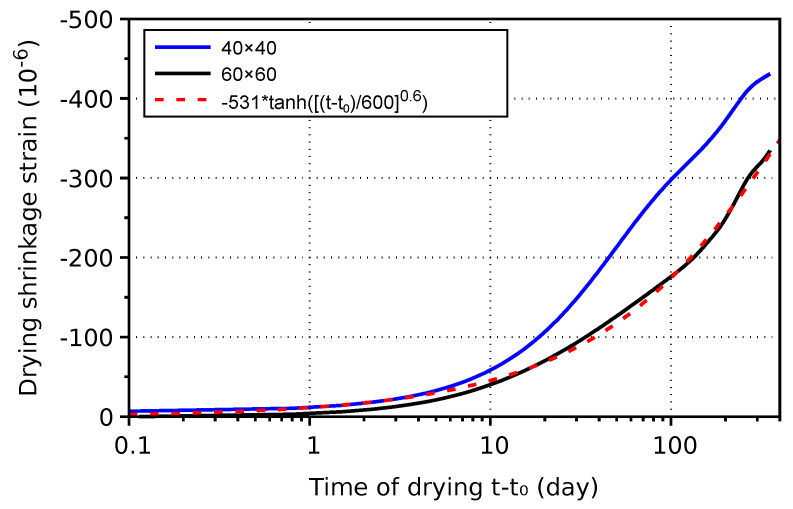
Drying shrinkage of concrete made from H-cement.

Drying shrinkage of concrete specimens is summarized in Figure 10. Ultimate shrinkage strain can be calculated from the ratio of concrete/mortar shrinkage at 350 days of 60×60 mm specimens, which yields −332/−452 · (−723) = −531. The approximation of concrete drying shrinkage strain reads

(5) $$\begin{array}{c} \varepsilon_{sh}\left(t\right)=-531\cdot 10^{-6}\tanh{\left(\frac{t-t_{0}}{600}\right)}^{0.6}, \end{array}$$

where drying would end in approximately 4 years.

#### 3.3.1. Basic Creep

**Figure 11:**
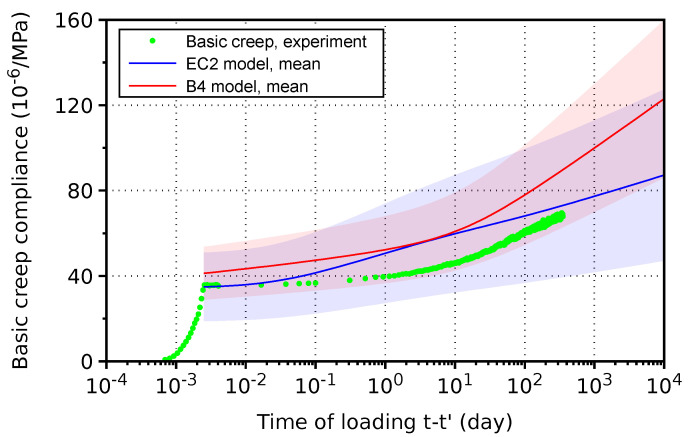
Basic creep of concrete made from H-cement. Filled area denotes standard deviation of each creep model.

Sealed concrete cylinders made of H-cement were placed in a rig and loaded at 14 days—see Figure 4 and Figure 11. The elastic strain immediately after loading was 36 · 10^−6^/MPa, corresponding to a static modulus of elasticity of 27.8 GPa. Creep measurements were not performed on the reference cement CEM II/B-S 32.5 R. Two models for creep, namely EC2 and B4, showed similar predictions as the experimental data. While the EC2 model corresponds better to early-age creep, the B4 model follows long-term creep closely. Previously, error coefficients of variation were determined from hundreds of experimental points, and the experimental data for H-cement-based concrete fall within the range of standard deviations [28].

#### 3.3.2. Total Creep

Total creep can be obtained from total deformation by subtracting drying shrinkage and autogenous shrinkage, with the latter being negligible. Drying shrinkage strain from a concrete prism of size 60×60 mm was used for estimating the behavior of the cylinder with a diameter of 150 mm. According to the B4 model, characteristic time is proportional to ${\left(k_{s}\cdot 2\cdot V/S\right)}^{2}$, where $k_{s}$ stands for the shape’s parameter, *V* represents the specimen’s volume and *S* its drying surface [19]. The prism 60 × 60 mm yields ${\left(1.25\cdot 2\cdot 15\right)}^{2}=1406$ mm^2^, while the cylinder yields ${\left(1.15\cdot 2\cdot 37.5\right)}^{2}=7439$ mm^2^. Thus, the ratio of drying kinetics is 1406/7439 = 0.189. The drying shrinkage strain of the cylinder corresponds to

(6) $$\begin{array}{c} \varepsilon_{sh}\left(t\right)=-531\cdot 10^{-6}\tanh{\left(\frac{0.189(t-t_{0})}{600}\right)}^{0.6}. \end{array}$$

**Figure 12:**
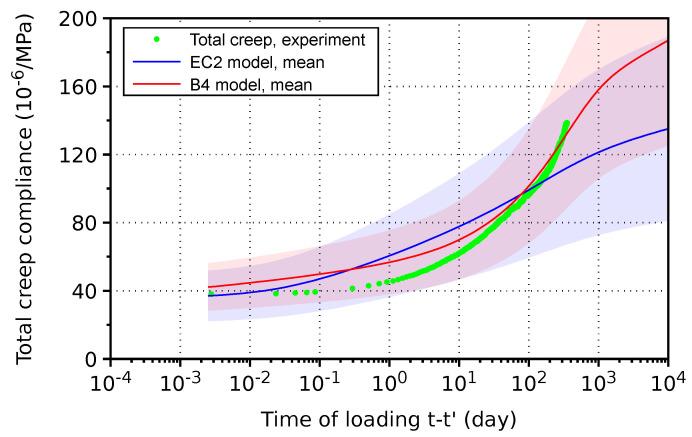
Total creep of concrete made from H-cement. Filled area denotes standard deviation of a model.

The compliance function in Figure 12 follows the mean values from the EC2 and B4 models. In all cases, the compliance falls within standard deviation bounds. The results are comparable with HVFAC behavior [29], which resembles 65% of the content of fly ash in H-cement.

## 4. Conclusions

This paper summarizes creep and shrinkage data of hybrid cement (called H-cement), mostly measured over a period of 350 days. The following conclusions were found:

- Autogenous shrinkage shows similar values up to 7 days between H-cement and CEM II/B-S 32.5 R pastes. After 7 days, H-cement shows smaller autogenous shrinkage.
- Drying shrinkage evolves similarly for mortars made from H-cement and CEM II/B-S 32.5 R cement. For the same shrinkage, H-cement loses approximately twice as much water. The reason for this likely stems from the coarser capillary pores and lower capillary pressure.
- The ultimate value of H-cement’s total shrinkage reached $-723\cdot 10^{-6}$ on mortar samples of size 25×25 mm. Similar values were achieved by a comparable mortar made from CEM II/B-S 32.5 R.
- Basic and total creep of H-cement concrete falls within the standard deviation margins of B4 and EC2 models for structural concrete. H-cement concrete exhibits similar total creep as high-volume fly ash concrete.
- Overall, the shrinkage and creep properties of H-cement showed similar values to comparable structural concretes, displaying potential for having low-carbon applications in the construction industry.

## Figures and Tables

**Table 1:** Characteristic oxide composition of materials.

	CaO	SiO_2_	Al_2_O_3_	Fe_2_O_3_	MgO	SO_3_	K_2_O	Na_2_O
Clinker (20%)	66.13	21.69	5.31	2.83	1.44	0.53	1.00	0.04
Fly ash (65%)	3.36	51.42	26.93	7.27	2.10	0.87	3.28	0.17
Slag (10%)	34.92	35.85	8.36	0.43	6.15	1.61	0.74	0.21
Alkali activator (5%)	-	-	-	-	-	55.78	-	43.02
H-cement	17.09	46.91	16.91	6.47	1.68	3.65	3.17	2.52
CEM II/B-S 32.5 R	54.87	26.33	6.06	2.56	4.08	2.34	0.67	0.26

**Table 2:** Strength evolution according to EN 196-1. H-cement used w/c = 0.40.

	w/c	Compressive Strength [MPa]			Flexural Strength [MPa]		
		2 Days	28 Days	90 Days	2 Days	28 Days	90 Days
H-cement	0.40	17.5 ± 3.0	36.5 ± 4.0	41.5 ± 3.0	3.5 ± 0.5	4.4 ± 0.4	9.0 ± 0.3
CEM II/B-S 32.5 R	0.50	13.4 ± 1.0	46.5 ± 0.9	60.0 ± 1.1	3.1 ± 0.3	8.9 ± 0.2	10.2 ± 0.3

**Table 3:** Mix design.

Item	Reference, Mortar (kg/m^3^)	H-Cement, Mortar (kg/m^3^)	H-Cement, Concrete (kg/m^3^)
H-cement		490	350
CEM II/B-S 32.5 R	516		
Water	232	221	158
Normal sand 0/2	1548	1470	
Fine aggregate 0/4 mm, Dobříň			835
Coarse aggregate 4/8 mm, Zbraslav			320
Coarse aggregate 8/16 mm, Zbraslav			713
Plasticizer (lignosulfonates)		3.4	2.4
Water/cement	0.45	0.45	0.45
Aggregate/cement	3.00	3.00	5.34

## Data Availability

Only the data published in this paper are available.
